# Bioinformation and Neutrino Communication

**DOI:** 10.6026/97320630018496

**Published:** 2022-06-30

**Authors:** Paul Shapshak

**Affiliations:** 1 Division of Infectious Diseases and International Health, Tampa General Hospital, Department of Internal Medicine, University of South Florida, Morsani College of Medicine, Tampa, FL 33606, USA

**Keywords:** Neutrino, communication, signal, detection, transmission, electromagnetism (EM), galaxy, bioinformation, life, astrobiology, astro-virology, extra-terrestrial intelligence (ETI), Kardashev-Dyson-Sagan (KDS), stratification, Dyson sphere, Drake-Sagan equation, star, planet, supernova, quasar, blazar, neutron star, pulsar, black hole, Standard Model, fermion, weak interaction, W+/W-/Zo bosons

## Abstract

Communications among civilizations may include self-descriptive bioinformation because pathogen dynamics exist in their astrobiology and astrovirology, which could become pathogenic upon actual contact. This information is of mutual benefit, if reciprocated. However, in contrast, the strategic counter-scenario of self-hidden civilizations is also discussed. Civilizations, including extra-terrestrial civilizations have been divided and stratified into three levels, using a wide non-linear logarithmic scale. The levels are based on their energy expenditures: level 1 is at 4x10^^^19 erg/sec; level 2 is at 4x10^^^33 erg/sec; and level 3 is at 4x10^^^44 erg/sec. Terrestrial civilization is currently below the entry level I. Particularly advanced civilizations, which are above the highest level, may engineer interstellar travel and could move their planets across interstellar distances. Communication among civilizations has always been of keen interest. In terms of ability to communicate among advanced civilizations, neutrinos may be used for galactic and inter-galactic communication, in addition to or instead of using electromagnetic radiation. Thus, at this juncture, deliberation and debate are essential to proceed with development of civilization and communication.

## Neutrino and Electromagnetic (EM) Communication:

Neutrino research provides an unparalleled window into fundamental particle cosmology. They have several characteristics that are under study, many of which extend beyond the Standard Model of particle physics. The primary effect they exhibit that has puzzled the field is that they have mass, which is not predicted within the Standard Model. A few neutrinos properties include the following. Cognate with the three negatively charged leptons (electron (e), muon (m), and tau (t)), neutrinos oscillate among three uncharged flavors (neutrino-e, neutrino-m, and neutrino-t). Neutrinos have very low energy and mass compared with the other elementary particles, and neutrinos have life-times akin to cosmological timescales [1-3]. Pasachoff, Saenz, and colleagues, since 1977, produced hypothesis, theory, and experimental data promoting the employment of neutrinos to detect potential communication signals from ETI civilizations. Neutrinos have small cross-sections and low-level weak interactions (via W+, W-, and Zo bosons) with fermion, lepton, and hadron matter. Consequently, these key features determine matter transparency to neutrinos. In contrast to neutrinos, EM radiation is less penetrating and is largely absorbed. Neutrinos have been produced artificially since the mid-20th century as by-products of fundamental particle and nuclear reactions, particle accelerators, radioactivity, nuclear reactors, and nuclear explosions. Neutrinos contribute 0.5% density of the universe and were produced naturally since the Big Bangas well as continuously throughout the duration of the existence of the universe. Naturally-produced neutrinos originate from stars, neutron stars, pulsars, quasi-stellar objects (QSOs), quasars, blazars, supernovae, black holes, and cosmic rays. Einstein predicted that light-path-geodesics are bent by gravitational fields as explained in the General Theory of relativity. This can be further observed as Einstein rings with multiple image projections, due to gravitational field lensing. This process applies to neutrinos, as well. Accordingly, neutrino sources may be magnified and intensified when neutrinos traverse intergalactic distances. Their paths, as cited for photons, are curved in space-time, due to gravitational effects that include lensing by galaxies, stars, and additional stellar objects. [3-12] The universe is expanding, resulting in photon increased wavelengths (red shift). Therefore, ETI-produced signals need to incorporate some form of 'timing' or 'life-time' correction codes, such that the recipients could establish distortions, perturbations, as well as space-time distances, which the signals traverse. In addition, the signals could embed entropy 'quantitation' of some sort, which would provide additional measures of accumulated disorder along their signal geodesics. Thus, necessary appropriate restructuring would be made by the recipients to translate signal information to the correct forms and frequencies for accurate representation and investigation. Such rectifications are required, much as stringed instruments are tuned to their correct harmonics and overtones. Furthermore, problems of transparency vs. types of opacities in interstellar and intergalactic space-time need to be addressed. Additionally, scientifically optimized cost vs. benefit analyses is required, because colossal disbursements of resources are incurred by communication recipients as well as by communication senders. Resolution of such economic complications is unavoidably tied to civilization resource and energy-use levels. [5,7,13, 14]

## Kardashev-Dyson-Sagan (KDS) stratification of civilization:

Several thousand years of recorded history have brimmed with speculations of extraterrestrial life and ETI. However, there have been no detections confirmed in accord with scientific methods. Nevertheless, in 1964, Kardashev hypothesized a type of organization of extraterrestrial civilizations, according to their estimated energy consumption. Kardashev designated three stratification levels and thus placed the search for extraterrestrials on a more scientific and technology-based footing, using measurables including energetics, technology, astronomy, and astrophysics. Sagan constructed an equation that allowed calculation of Kardashev intermediate echelons among the three levels. Remarkably, estimation of the energy utilization of current terrestrial technology placed our current development below Stage I in Kardashev's stratification system. Using these constraints, it will take an additional 327 years for human civilization to reach Stage I. [15- 17] Using the EM paradigm, Kardashev calculated energy production and message transmission characteristics. Analysis of cosmic EM signal vs. noise parameters, Kardashev calculated signal transmission possibilities, estimating required energy levels for communicating civilizations. His type II category civilizations could transmit at a power of 4x10^33 erg/sec. At a range of 100,000 light-years, 3x10^9 erg/sec could be received, at 10 million light-years, 3x10^5 erg/sec could be received, and at 10 billion light-years, information reception would be scarcely possible. However, type III civilizations could transmit at a power of 4x10^44 erg/sec. At a range of 100,000 light-years, 2.4x10^15 erg/sec could be received, at 10 million light-years, 2.4x10^13 erg/sec could be received, and at 10 billion light-years, information reception could be 3x10^10 erg/sec. Kardashev stated that the interstellar medium could exhibit optimal transparency EM frequency at 10^9-10^11 Hz. Additionally, Kardashev stated that with 10^11 stars in our galaxy and with 10^10 observable galaxies (in 1964), it appeared unlikely that our presence was the only one in the universe. Further, he observed that interstellar communication with other civilizations was possible using EM radio-astronomy. He also proposed that CTA-21 and CTA-102 were possible signal sources and that further observations and research were needed. CTA-21 has a red-shift of 0.907, which is 12.489 billion light years away.CTA-102 is a quasar-blazar galaxy with a 1.037 redshift, which is 14.279 billion light years away. Both have highly complex modulated spectra. [15,16]Additionally, very advanced Kardashev technological civilization may modify such massively energetic beacons and thereby transmit information. Possibly, self-revelatory civilizations, to reduce dangers of conflict, may provide bioinformation including their biochemistry and biology, potential infectious diseases, incorporating viruses, as well as 'psychological' profiles in their transmissions. The pros and cons of self-revelatory 'bioinformation' need further analysis. [15,16] Dyson proposed that very advanced civilizations could construct energy catchments around their parent stars to harness energy at superior efficiency and augment acceleration of their technological capabilities and development. This would thereby increase their level in the Kardashev stratification system. Many star types have been proposed as potentially surrounded with spherical placement of energy-seizing apparatus. Potential energy sources include black holes, neutron stars, red dwarfs, etc. However, leaked, unused, and waste energy from Dyson spheres is anticipated to be in the infrared. Such targets are weak with which to initiate studies. (Human engineering and power consumption, on the average, has 60-70% efficiency of energy delivery). Extraterrestrials, presumably, would have improved efficiency above that. A further level in the KDS energetics technology stratification system, would be for civilizations to develop engineering and energy capabilities to propel their planets into escape velocity from their solar systems. This would be at pre-eminent levels of accomplishment. [18-22]

## Bioinformation in neutrino dynamics, astrobiology, and astro-virology:

The possible influence of neutrino dynamics on planetary astrobiology/exobiology is a recent addition to origin of life research. In point, neutrino chirality and flavor may influence molecular chirality selection and discriminatory mechanisms during the origin and development of life. Such processes could occur, depending on the proximity of neutrino sources, such as their suns, other stars, neutron stars, dwarfs, supernovae, pulsars, quasars, and blazars. Whether neutrino dynamics influenced the terrestrial origin of life in any respect is under investigation. [23,24] During prior millennia, infectious diseases had profound deadly consequences vis-a-vis human and animal health. Moreover, until the 20th century, there was little comprehension of the numerous unanticipated terrestrial infectious disease epidemics and pandemics. Thus, it is with alarmed concern that this article opines possible toxic and pathogenic risks due to potential contact with extraterrestrial astrobiologies and astro-virologies. In addition, concomitant with human expansion and extraterrestrial travel and exploration, micro-organism passengers can mutate in the various target environments. Consequently, upon returning to Earth, novel and unforeseen epidemics and pandemics could be the upshot.

## Safety analysis:

Analysis of several diverse research fields syndicate global consequences that were previously unanticipated by each alone. Jointly and individually, the sundry arenas mentioned in this article have bearings on human health, safety, communication, technological development, and survival. Prior cultures and civilizations analyzed such complications with some degree of histo-riographic accuracy. In 411 BCE, in Athens, Thucydides published one of the earliest history books, 'The History of the Peloponnesian Wars'. He discussed the problems faced by prosperous city-states becoming victims to aggressive predatory city-states. Consequently, Thucydides advocated concealment as a strategy to avoid predation and annihilation. However, about 2,393 years later, Sagan and colleagues were less pessimistic about such situations, with respect to ETI and terrestrial civilizations. Based on the Drake-Sagan equation and possible rates of ETI civilization galactic diffusion theory, probability scenarios were calculated among ETIs at differing relative times. It may be inferred that consequently, an inferior ETI civilization would have negligible sway on the contact outcome and would be utterly at the mercy of the more developed superior ETI civilization. Similarly, terrestrial civilization would likely be dominated by ETI civilizations that happened upon our solar system and the Earth [25,26]. Nevertheless, these disparate views, ranging from Thucydides to Sagan may not be absolute. In contrast, mathematician John Nash, who won the Economic Sciences Nobel Memorial Prize in 1994, analyzed conflict and competition? He ascertained that within specific contexts, equipoise points or states of equilibrium exist. This mathematically constructed scenario portends the possibility of conflict avoidance, if the players in such contact situations were logically and constructively inclined. [27, 28]

## Conclusions:

In conclusion, undoubtedly, far-reaching impacts on terrestrial civilization will occur, if we continue attempts to communicate with extraterrestrial ETI civilizations, which could result in contact. The outcomes portend being illogical games of dice. The gamut of connection scenarios includes neutrino and EM communications and depends on fields embracing particle physics, astrophysics, astrobiology, astro-virology, and artificial intelligence. We may add that revelatory civilizations could provide crucial bioinformation in their communications, which would be important in any possible actual interactions. From a Bioinformation point of view, contacts would then not be proceeding completely blindly, but nonetheless would remain dicey at best [29,30].
